# Conformational Flexibility Differentiates Naturally Occurring Bet v 1 Isoforms

**DOI:** 10.3390/ijms18061192

**Published:** 2017-06-03

**Authors:** Sarina Grutsch, Julian E. Fuchs, Linda Ahammer, Anna S. Kamenik, Klaus R. Liedl, Martin Tollinger

**Affiliations:** 1 Institute of Organic Chemistry & Center for Molecular Biosciences Innsbruck (CMBI), University of Innsbruck, Innrain 80/82, A-6020 Innsbruck, Austria; sarina.grutsch@uibk.ac.at (S.G.); linda.ahammer@uibk.ac.at (L.A.); 2 Institute of Inorganic and Theoretical Chemistry & Center for Molecular Biosciences Innsbruck (CMBI), University of Innsbruck, Innrain 80/82, A-6020 Innsbruck, Austria; julian.fuchs@uibk.ac.at (J.E.F.); anna.kamenik@uibk.ac.at (A.S.K.)

**Keywords:** allergens, proteolytic processing, allergic sensitization, allergen structure, flexibility

## Abstract

The protein Bet v 1 represents the main cause for allergic reactions to birch pollen in Europe and North America. Structurally homologous isoforms of Bet v 1 can have different properties regarding allergic sensitization and Th2 polarization, most likely due to differential susceptibility to proteolytic cleavage. Using NMR relaxation experiments and molecular dynamics simulations, we demonstrate that the initial proteolytic cleavage sites in two naturally occurring Bet v 1 isoforms, Bet v 1.0101 (Bet v 1a) and Bet v 1.0102 (Bet v 1d), are conformationally flexible. Inaccessible cleavage sites in helices and strands are highly flexible on the microsecond-millisecond time scale, whereas those located in loops display faster nanosecond-microsecond flexibility. The data consistently show that Bet v 1.0102 is more flexible and conformationally heterogeneous than Bet v 1.0101. Moreover, NMR hydrogen-deuterium exchange measurements reveal that the backbone amides in Bet v 1.0102 are significantly more solvent exposed, in agreement with this isoform’s higher susceptibility to proteolytic cleavage. The differential conformational flexibility of Bet v 1 isoforms, along with the transient exposure of inaccessible sites to the protein surface, may be linked to proteolytic susceptibility, representing a potential structure-based rationale for the observed differences in Th2 polarization and allergic sensitization.

## 1. Introduction

In Western countries, 25% of the population suffer from allergies, and among the most abundant ones are those related to tree-pollen [1]. More than 90% of tree pollen-allergic patients react to allergens from the European white birch (*Betula verrucosa*) and 60% or more of birch pollen allergic patients react exclusively to the major birch pollen allergen Bet v 1 [2,3]. This protein, which belongs to the pathogenesis-related class 10 protein family (PR-10), acts as a trigger for type I allergies [4]. Bet v 1 folds into a seven-stranded, anti-parallel β-sheet embracing a long helix at the C-terminus of the protein, along with two consecutive short helices that form a V-shaped support for the C-terminal helix. In nature, Bet v 1 comprises a complex mixture of different isoforms [5,6], of which more than twenty are currently listed by the IUIS Allergen Nomenclature Sub-Committee (www.allergen.org). Bet v 1 isoforms at www.allergen.org share between 69.8% and 99.4% sequence identity and exhibit only minor differences of their three-dimensional structures, with backbone pair-wise root-mean-square deviation (r.m.s.d.) values between 1.5–2 Å [7]. Despite their high structural similarities, however, Bet v 1 isoforms are known to have drastically different allergenic properties [8]. The most abundant isoform, Bet v 1.0101 (Bet v 1a according to former nomenclature), which constitutes around 35% of total Bet v 1 in pollen grains [8], acts as a sensitizing agent inducing a Th2-biased immune response, as characterized by aberrantly high IgE levels [9,10]. This is contrasted by Bet v 1.0102 (Bet v 1_T7I/F30V/S57N/I91V/S112C/I113V/D125N_, formerly Bet v 1d), which constitutes around 10% of total Bet v 1 in pollen grain. Bet v 1.0102 is known to induce only a minimal IgE response, even though it shares 95.6% sequence identity with Bet v 1.0101. As a matter of fact, Bet v 1.0102 appears to induce a protective immune response, rather than acting as a sensitizing agent [9].

One of the key steps in the sensitization process against Bet v 1 is the proteolysis of the protein backbone in the endolysosomal compartment and the subsequent presentation of the antigen-derived peptide fragments on the surface of antigen presenting cells by major histocompatibility complex (MHC) class II molecules [11]. A fundamental requirement for proteolysis is the accessibility of backbone cleavage sites to proteases. For Bet v 1, four early cleavage sites have been identified [12,13]. Intriguingly, however, these early cleavage sites in the Bet v 1 backbone are hydrogen bonded in secondary structure elements or tight turns and, hence, are not accessible to endosomal proteases per se. Based on these observations, it has been hypothesized that the conformational flexibility of the Bet v 1 protein backbone could be decisive and critical for proteolytic processing [13]. Generally, proteins possess not only a single, static structure, but also consist of a versatile ensemble of interconverting conformers with different features [14,15]. Structural variations can range from local, small-scale fluctuations of bond lengths, angles, and dihedrals, all the way to large-scale changes of secondary structure elements and re-arrangements of the entire protein fold. As a result, potential cleavage sites in secondary structures could be transiently exposed to the protein surface, rendering them accessible to proteolytic enzymes.

Interestingly, in vitro degradation assays of Bet v 1.0101 and Bet v 1.0102 using endolysosomal preparations from dendritic cells as the protease source, along with mass-spectrometric analyses, revealed different degradation rates for these two isoforms, but very similar proteolytic fragmentation patterns [12]. More (Bet v 1.0101) and less (Bet v 1.0102) immunogenic isoforms of Bet v 1 thus appear to differ regarding the rates at which peptide bonds are cleaved, rather than the sites of fragmentation. Employing the isolated cysteine protease cathepsin S, which is likely involved in the initial steps of Bet v 1 processing in antigen presenting cells [12], Freier et al. further showed that in Bet v 1.0102 the early proteolytic cleavage sites are more easily accessible to protease binding than in Bet v 1.0101 [13]. Based on these results, it was postulated that the isoform-specific conformational flexibility of Bet v 1 could be a key parameter that determines the efficacy of proteolytic fragmentation. Early cleavage sites in Bet v 1.0102 might be more efficiently bound and cut by proteases due to a higher level of conformational flexibility.

Here, we provide a quantitative, comparative description of the conformational flexibilities of Bet v 1.0101 and Bet v 1.0102 by combining nuclear magnetic resonance (NMR) experimental data with computer simulations. The conformational flexibilities of these two proteins were probed by a variety of NMR relaxation experiments and molecular dynamics simulations. Furthermore, NMR hydrogen-deuterium exchange experiments were used to measure the solvent accessibilities of the backbone amides of Bet v 1.0101 and Bet v 1.0102 in a site-specific manner. Our results indicate that the differential conformational flexibilities of Bet v 1 isoforms are a protein-inherent feature that may represent a direct linkage to the observed differences in their allergic sensitization potential.

## 2. Results

### 2.1. Microsecond-Millisecond Conformational Flexibility

**Figure 1:**
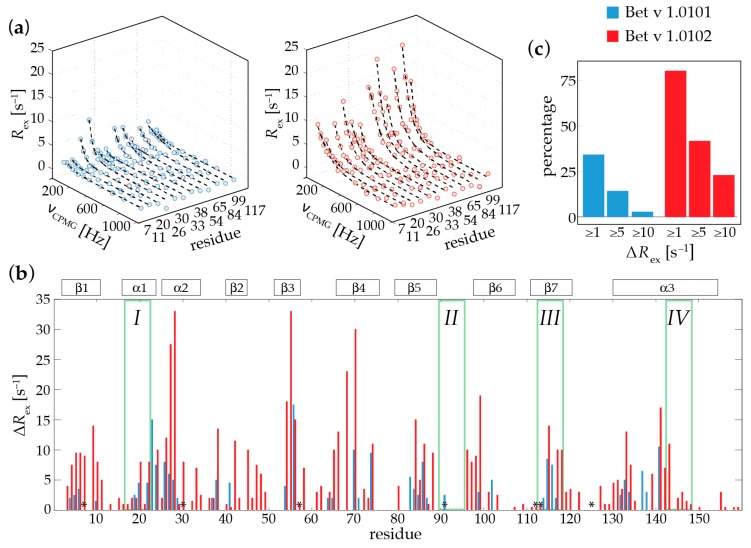
Experimental NMR microsecond-millisecond conformational flexibility in Bet v 1 isoforms. (**a**) Backbone amide ^15^N relaxation dispersion profiles for Bet v 1.0101 (**blue**, **left**) and Bet v 1.0102 (**red**, **right**), recorded at 800 MHz, 298 K, pH 8.0. Experimental data (circles) are shown for a subset of 12 representative amino acid residues in both isoforms (Thr7 (Ile7 in Bet v 1.0102), Ser11, Lys20, Gly26, Phe30 (Val30 in Bet v 1.0102), Val33, Ile38, Lys54, Lys65, Ser84, Ser99, Ser117), along with best-fit curves (dashed lines). Microsecond-millisecond time scale transitions between different conformers are manifest as non-flat relaxation dispersion profiles (*R*_ex_ > 0); (**b**) Site-resolved amplitudes, Δ*R*_ex_ = *R*_ex_ (ν_CPMG_ = 1000) − *R*_ex_ (ν_CPMG_ = 0), of the relaxation dispersion profiles for Bet v 1.0101 (**blue**) and Bet v 1.0102 (**red**). Amino acid positions that are different in Bet v 1.0101 and Bet v 1.0102 are indicated by asterisks (*), and early proteolytic cleavage sites *I*–*IV* according to Freier [13] are shown in green. Secondary structure elements (α1–α3 and β1–β7) are shown as defined by Gajhede et al. [17]; (**c**) Proportions of backbone amides for which Δ*R*_ex_ values in Bet v 1.0101 (**blue**) and Bet v 1.0102 (**red**) are equal to or greater than 1, 5 and 10 s^−1^, respectively.

To probe the conformational flexibilities of different Bet v 1 isoforms, we performed backbone amide ^15^N relaxation dispersion (RD) experiments, which monitor transitions between different conformers occurring on the microsecond-millisecond time scale. Such processes manifest themselves in contributions to the experimentally observed line-width of NMR resonances, which can be studied in a quantitative manner by RD-NMR spectroscopy [16]. Figure 1a shows representative backbone amide ^15^N relaxation dispersion data of Bet v 1.0101 and Bet v 1.0102 for a subset of 12 amino acid residues in these two proteins. In these plots, non-flat relaxation dispersion profiles are an experimental footprint of the transitions between different conformers within microsecond-milliseconds. This is contrasted by flat RD profiles, which are expected for backbone amides that are rigid on this time scale.

The backbone amide RD-NMR data of the two Bet v 1 isoforms point towards a flexible protein scaffold. This conformational flexibility is not limited to loops or turns and solvent exposed parts of the Bet v 1 fold. Rather, the flexible and dynamic parts of the protein backbone are fairly well distributed along the entire protein sequence, with some dynamic hot spots in secondary structure elements, including the two short helices α1 (which contains the early proteolytic cleavage site *I*) and α2, as well as residues that are embedded in the central β-sheet (strands β3, β4, and β7, containing cleavage site *III*) and the N-terminal and central parts of the long C-terminal helix α3 (cleavage site *IV*).

In addition, it is evident from Figure 1 that the two isoforms vary substantially regarding their flexibilities: When compared to Bet v 1.0101, the isoform Bet v 1.0102 displays dispersion profiles of a significantly larger amplitude, which is indicative of a higher level of conformational flexibility in this protein (Figure 1a). Sequence resolved amplitudes of the RD profiles of Bet v 1.0101 and Bet v 1.0102 are illustrated in Figure 1b. The amino acids that show increased values of Δ*R*_ex_ in Bet v 1.0102 do not coincide with the residues that are different in the two isoforms (Figure 1b), which could be expected for the local de-stabilization of the protein backbone in these parts of the protein. Rather, these data suggest that in Bet v 1.0102 the entire protein scaffold is more flexible than in Bet v 1.0101. Figure 1c explicitly compares the proportion of backbone amides that are flexible in these two isoforms, revealing that in Bet v 1.0102 a larger fraction of the protein backbone displays microsecond-millisecond time scale flexibility when compared to Bet v 1.0101. Roughly one-third (34%) of all backbone amides in Bet v 1.0101 have non-flat RD profiles, while for the remainder of the protein backbone, the dispersion profiles are flat (*R*_ex_ < 1 s^−1^). In contrast, in Bet v 1.0102, significantly more RD profiles (approx. 80% of all residues) are non-flat, respectively. A total of 26% of all backbone amides in Bet v 1.0102 have *R*_ex_ values of 10 s^−1^ or more, indicating the extensive microsecond-millisecond conformational flexibility of its protein backbone, while only 3% of all backbone amides in Bet v 1.0101 fall into this category (Figure 1c). Taken together, the experimental RD-NMR data thus directly show that Bet v 1.0101 and Bet v 1.0102 have measurably different conformational flexibilities on the microsecond-millisecond time scale.

### 2.2. Molecular Dynamics Simulations

**Figure 2:**
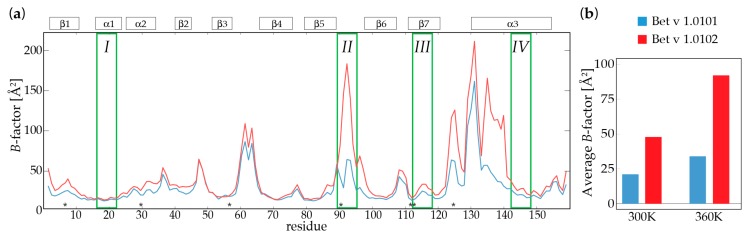
Molecular dynamics simulations of nanosecond-microsecond conformational flexibility in Bet v 1 isoforms. (**a**) Site-specific *B*-factors of Bet v 1.0101 (**blue**) and Bet v 1.0102 (**red**) derived from structural ensembles of microsecond molecular dynamics (MD) simulations at 300 K. Flexibility on the nanosecond-microsecond time scale leads to elevated *B*-factors. Secondary structure elements are indicated on top. Amino acid positions that are different in Bet v 1.0101 and Bet v 1.0102 are indicated by asterisks (*) and the early proteolytic cleavage sites *I*–*IV* are shown in green. (**b**) Comparison of average *B*-factors of Bet v 1.0101 (**blue**) and Bet v 1.0102 (**red**) at MD simulation temperatures of 300 and 360 K, respectively.

To further probe the conformational flexibilities of these two Bet v 1 isoforms, we performed molecular dynamics (MD) simulations of Bet v 1.0101 and Bet v 1.0102 at two temperatures, 300 and 360 K (Figure 2). Site-specific *B*-factors derived from the MD structural ensembles show that both isoforms have relatively rigid secondary structure elements on the nanosecond-microsecond time scale. In contrast, the short loops between strands β3 and β4 and strands β5 and β6 (containing the early proteolytic cleavage site *II*), the long loop between strand β7 and helix α3, and the N-terminal half of the long helix α3 display above average nanosecond-microsecond time scale flexibilities, with site-specific *B*-factors exceeding 50 Å^2^. These structural elements, which are partly solvent-exposed, surround the large entrance ε1 that enables the access of ligand molecules to the internal cavity of Bet v 1 [7,18].

It is of particular interest that at 300 K, we already observe differences in the ensemble-averaged structure and dynamics between the two isoforms. Conformational flexibilities measured by *B*-factors derived from the MD structural ensembles are considerably higher in Bet v 1.0102 when compared to Bet v 1.0101. More specifically, the loop region between the strands β5 and β6 (cleavage site *II*), the N-terminal part of helix α3, and the loop between β7 and α3 have markedly different flexibilities in the two isoforms. We find all these regions to be more flexible in Bet v 1.0102 compared to Bet v 1.0101, while only minor differences are observed for the central β-sheet of Bet v 1. Figure 2b compares the MD-derived average *B*-factors of the two isoforms, indicating a less flexible fold for Bet v 1.0101 when compared to Bet v 1.0102. The computational data further show that Bet v 1.0101 has a compact fold with a high secondary structure content and a strong internal hydrogen bonding network, while in Bet v 1.0102, the average number of hydrogen bonds in the MD structural ensemble is considerably smaller (see Table 1). In addition, the solvent accessible surface area (SASA) in Bet v 1.0102 is slightly elevated.

Additional simulations were performed at 360 K to explore further regions of the proteins’ conformational space and to overcome the sampling barriers. In the 360 K simulation, Bet v 1.0101 is again the more rigid protein, with a lower flexibility and stronger intramolecular interactions when compared to Bet v 1.0102. At this elevated temperature, both proteins show decreased compactness, hydrogen bonding, and secondary structure content, whereas the internal flexibility as measured by *B*-factors, is increased (Table 1). Taken together, both MD simulations reflect the same overall trend and clearly show a distinct difference in the conformational flexibilities of the two Bet v 1 isoforms on the nanosecond-microsecond time scale, with Bet v 1.0101 being the more rigid isoform, while Bet v 1.0102 populates a broader conformational ensemble.

### 2.3. Backbone Amide Hydrogen-Deuterium Exchange

Conformational flexibility can result in the transient exposure of backbone amides to solvent water. Since experimental NMR and computational MD data consistently indicate that Bet v 1.0102 is more flexible than Bet v 1.0101, we experimentally probed the accessibility of backbone amides to solvent water by NMR hydrogen-deuterium exchange measurements [19,20]. In these experiments, the exchange of backbone amide hydrogen to deuterium is measured by dissolving protonated protein in D_2_O and following the disappearance of the amide NH resonances in a series of two-dimensional NMR spectra. Backbone amide hydrogen atoms are protected from exchange as long as they are hydrogen-bonded or buried within the protein, as is the case for most amide hydrogen atoms in folded proteins. Only when, by conformational flexibility, an amide proton becomes solvent exposed can it be replaced by a deuteron. The measurement of hydrogen-deuterium exchange thus provides information on the exposure of backbone amides to solvent in a site-specific manner.

**Figure 3:**
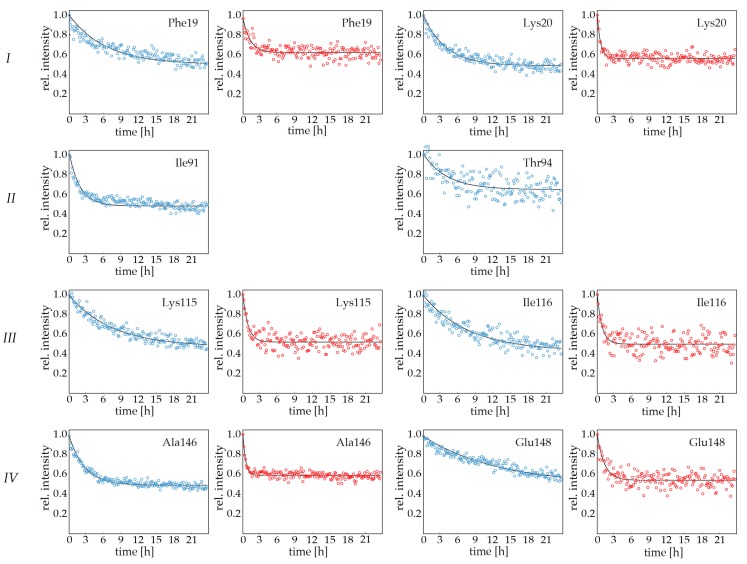
Experimental NMR backbone amide hydrogen-deuterium exchange in Bet v 1 isoforms. Representative SOFAST data for residues in the early proteolytic cleavage sites *I*–*IV* (**top** to **bottom**). Coloring scheme: (**blue**) Bet v 1.0101; (**red**) Bet v 1.0102. Best-fit exponential curves are shown in black. In Bet v 1.0102, experimental data for backbone amides surrounding cleavage site *II* could not be obtained due to very fast hydrogen-deuterium exchange. Data for both isoforms were recorded at 283 K, pH 8.0.

For many backbone amide protons in Bet v 1.0102 and, to a much lesser extent, in Bet v 1.0101, exchange with D_2_O was too fast to be observed by standard two-dimensional techniques at 298 K, indicating a remarkably high level of solvent exposure in both isoforms. To increase the fraction of observable backbone amides, we performed all experiments at a reduced temperature (283 K) employing the SOFAST (band-selective optimized flip-angle short transient) rapid data acquisition technique [21]. Using these experiments, two-dimensional ^1^H^15^N correlation spectra could be obtained in 8.5 min, which enabled us to obtain reliable hydrogen-deuterium exchange data for the majority of all observable backbone amide groups in these proteins. Figure 3 shows a comparison of the experimental data of these isoforms for amino residues that are located at (or close to) the early proteolytic cleavage sites.

For Bet v 1.0101, the hydrogen-deuterium exchange of backbone amides in secondary structure elements (e.g., Phe19 and Lys20 in helix α1, Lys115, and Ile116 strand β7, Glu148) is complete after a few hours, and even faster exchange rates are found for backbone amides in loops and turns (e.g., Ile91 and Thr94). These data indicate that backbone amides in all four early proteolytic cleavage sites *I*–*IV* are indeed transiently exposed to solvent water and capable of hydrogen exchange with D_2_O, despite the fact that many of these backbone amides are hydrogen bonded and buried in the protein interior in Bet v 1 crystal structures. Most significantly, the experimental data for Bet v 1.0102 shows that this isoform exchange is accelerated by a factor of 8–10 under identical conditions. This is the case for all backbone amides in Bet v 1.0102, ruling out local effects involving only the seven amino acids that are different in the two isoforms (the intrinsic susceptibility to exchange can be influenced by amino acid substitutions up to two positions away from a particular residue [22]). Notably, for a number of residues in Bet v 1.0102, hydrogen-deuterium exchange in solvent exposed loops is too fast to be detected in our SOFAST experimental setup, even at 283 K, although we did observe nearly all backbone amide resonanced in NMR spectra. This is the case for Val91 and Thr94, which are located in a loop connecting secondary structure elements, indicating the higher solvent accessibility of residues that are close to the protein surface. The systematic difference between the two Bet v 1 isoforms at both temperatures clearly shows that the accessibility of backbone amide sites to solvent water and the susceptibility to hydrogen-deuterium exchange is substantially enhanced in Bet v 1.0102.

## 3. Discussion

Antigen presenting cells (APC’s) process antigens by numerous endolysosomal proteases, resulting in only modest cleavage specificities at the level of the primary sequence [23]. However, peptide bonds within secondary structure elements are poorly accessible to proteases due to the framework of stabilizing hydrogen bonds and steric hindrance, so that the proteolytic cleavage of antigens tends to occur in loop regions and surface exposed segments. In addition, most proteases bind their substrates in a fairly extended conformation, where side chains can favorably interact with the active site of the protease, requiring a certain degree of flexibility for optimal binding [24].

**Figure 4:**
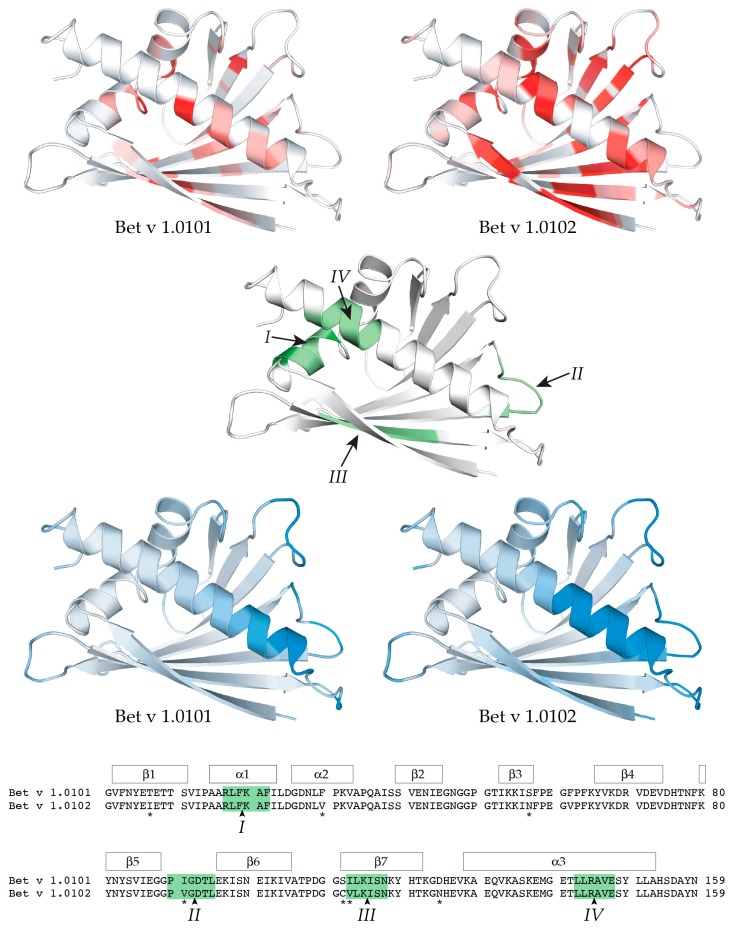
Conformational flexibilities of Bet v 1 isoforms Bet v 1.0101 and Bet v 1.0102. Top: Site-specific microsecond-millisecond time scale flexibilities (*R*_ex_ values) derived from NMR relaxation dispersion experiments, colored from **red** (high flexibility) to **white** (low flexibility). Bottom: Site-specific nanosecond-microsecond flexibilities (B-factors) from MD simulations, colored from **blue** (high flexibility) to **white** (low flexibility). For both isoforms, identical color thresholds were used. Center: Early proteolytic cleavage sites *I*–*IV* according to Freier et al. [13]. Protease recognition sites between the non-primed site P3 and the primed site P3’ are shown in green (site *I*: Arg17-Phe22, site *II*: Pro90-Leu95, site *III*: Ile113-Asn118, site *IV*: Leu143-Glu148) and the scissile peptide bonds are marked by black arrows. Bottom: Sequence alignment of Bet v 1.0101 and Bet v 1.0102 obtained with Clustal Omega [25]. Secondary structure elements (Bet v 1.0101) are indicated and early proteolytic cleavage sites *I*–*IV* are displayed in **green**. Sequence differences between Bet v 1.0101 and Bet v 1.0102 are marked with asterisks.

In Bet v 1, most of the early proteolytic cleavage sites are located in secondary structure elements, with their backbone amides being hydrogen bonded to other amides nearby. Two of the Bet v 1 early cleavage sites are found in helices: Cleavage site *I* is located in the middle of the short helix α1, which forms part of the V-shaped support for the C-terminal part of helix α3, and cleavage site *IV* is located in the center of helix α3 (Figure 4). Protein helices are considered particularly poor substrates for proteolysis [24], since helices represent a fairly compressed structural motif with backbone amides being involved in an intramolecular pattern of hydrogen bonds. In helices, only the side chains are available for intermolecular interactions with a protease, and peptide bonds have to be transiently exposed to the surface to be available for proteolytic cleavage. The early cleavage site *III* is located within the central β-sheet, in strand β7, away from the edges of the seven-stranded antiparallel sheet. All backbone amides in this cleavage site are inter-strand hydrogen bonded to adjacent β-strands (β1 or β6) and the peptide bonds are inaccessible to the solvent and proteases. While proteases preferably bind substrates in extended conformations, breakage of the β-sheet inter-strand hydrogen bonds is required to form a single strand that is susceptible to proteolysis [24]. Only site *II* in the loop between strands β5 and β6 is located close to the protein surface (Figure 4). Its scissile peptide bond between Gly92 and Asp93, however, is embedded in a tight β-turn with the backbone carbonyl oxygen of Gly92 being hydrogen bonded to the backbone amide of residue Leu95, rendering this peptide bond only partly accessible to proteolytic enzymes.

Figure 4 compares the conformational flexibilities of the two isoforms Bet v 1.0101 and Bet v 1.0102 to their early proteolytic cleavage sites *I*–*IV*. Microsecond-millisecond time scale flexibility, as probed by relaxation dispersion NMR, involves a substantial proportion of amino acids in secondary structure elements, but also includes turns and loops. Molecular dynamics-derived nanosecond time scale flexibility is particularly pronounced for mobile loops connecting secondary structures and parts of the C-terminal helix α3. Notably, it is evident from Figure 4 that the conformational flexibility in both Bet v 1.0101 and Bet v 1.0102 covers (but is not limited to) the early proteolytic cleavage sites in these proteins. Three of the early proteolytic cleavage sites (*I*, *III*, and *IV)* are located in parts of the protein that are flexible on the microsecond-millisecond time scale (secondary structure elements α1, β7, and α3, respectively), while cleavage site *II* in the hydrogen bonded tight turn between strands β5 and β6 displays significantly above average nanosecond-microsecond time scale dynamics.

NMR analysis has shown that microsecond-millisecond time scale conformational flexibility in Bet v 1.0101 is accompanied by an increase in the radius of gyration, indicating a measurable loss of compactness and loosening of the protein scaffold [26]. Differences between the involved structures are small, ruling out segmental unfolding of the protein backbone [26]. Rather, the structural flexibility on this time scale is reminiscent of “conformational breathing” of the protein scaffold [27], as reported for various lipid binding proteins and lipocalins [28,29,30]. These carrier proteins have topologies that are similar to Bet v 1, consisting of eight- or ten-stranded antiparallel β-sheets and one or two helices around an internal cavity. Conformational breathing, i.e., extensive but largely uncorrelated flexibility involving a significant portion of the protein scaffold, is believed to be related to the mechanism by which these proteins allow ligand entry to the interior cavity [29,31]. In Bet v 1, microsecond-millisecond conformational breathing includes the central β-sheet and the three α-helices, highlighting the inherently flexible and heterogeneous nature of these structural elements, despite the presence of numerous hydrogen bonding interactions in the static structure. Such motions may well lead to the transient exposure of potential cleavage sites to the protein surface and facilitate intermolecular interactions with substrate binding sites of proteases.

Cleavage site *II*, on the other hand, displays significant nanosecond-microsecond time scale dynamics in molecular dynamics simulations. The relatively high flexibility of the loop between strands β5 and β6 extends to other surface exposed loops and the N-terminal half of helix α3, which cluster around the largest entry site to the internal cavity of Bet v 1. It has been previously noted that enhanced backbone flexibility in molecular dynamics studies is a key feature of proteolytic cleavage sites [32]. Increased nanosecond time scale flexibility around entry gates has also been reported in experimental and computational studies of lipid binding proteins, and a potential role of these motions for ligand entry to the protein’s internal cavity is being disputed [31,33]. Our molecular dynamics simulations do not indicate any local unfolding of the N-terminal half of helix α3 on the nanosecond time scale. However, the β5/β6 tight turn is disrupted in the MD ensemble and the hydrogen bond between the CO of 92 and NH of 95 is present in only 8% (Bet v 1.0102) and 17% (Bet v 1.0101) of the structures. From our observations, it is thus tempting to speculate that in Bet v 1, rapid, nanosecond-microsecond conformational dynamics of the polypeptide backbone facilitate interactions with proteases in surface exposed loops, while the loosening of hydrogen bonded secondary structure elements, which is required for the proteolytic attack of these peptide bonds, occurs on the microsecond-millisecond time scale.

In vitro degradation assays using dendritic cells as protease sources [12] or employing the cysteine protease cathepsin S [13] indicate that Bet v 1.0102 is measurably faster degraded than Bet v 1.0101, implying that there is an increased exposure or flexibility of its peptide bonds to solvent. In addition, processing by proteases in endosomal compartments is strongly pH dependent [13,34]. Endocytosed proteins are subject to acidification from pH 7 to pH 4, and variations in the proteolytic susceptibility of Bet v 1 as it passes through the pH gradient during endosomal maturation are key for their immunogenicity. In pH-dependent studies [13], it was shown that non-sensitizing Bet v 1.0102 is readily bound by protease and processed at pH > 5.5, resulting in the rapid formation of large amounts of peptide fragments and, consequently, protective Th1 polarization and a protective immune response. This is contrasted by Bet v 1.0101, which is degraded more slowly, providing a continuous supply of peptide fragments for presentation to MHC class II molecules at a low dose, which favors Th2 polarization and allergic sensitization. Due to the limited solubility of Bet v 1 isoforms close to their isoelectric points (the predicted pI values of Bet v 1.0101 and Bet v 1.0102 are 5.4 and 5.8, respectively), all NMR experimental data in our study were recorded at pH 8.0. Our data indicate that even at pH values outside the pH range that is critical for antigen processing, conformational flexibility in Bet v 1 isoforms is extensive and significant differences between structurally homologous isoforms exist. Our data may thus provide a first structure-based clue for the observed differences of the proteolytic behavior of these two proteins: NMR experimental and MD computer simulation data consistently show that Bet v 1.0102 is conformationally more flexible than Bet v 1.0101, including all four early proteolytic cleavage sites. Conformational flexibility can enable access to the peptide bond and facilitate interactions with proteases and enhance cleavage [35]. The higher backbone flexibility in Bet v 1.0102 may thus represent a mechanism that enables the more efficient (fast) proteolytic cleavage of this isoform by exposing protein peptide bonds in a larger fraction of molecules. In cases where conformational flexibility involves the breaking of hydrogen bonds or the exposure of backbone amides to solvent, increased hydrogen-deuterium exchange rates are to be expected. Our NMR hydrogen-deuterium exchange data indeed imply that the percentage of backbone amides that are solvent exposed in Bet v 1.0102 is considerably higher than in Bet v 1.0101.

Taken together, our studies reveal that (i) early proteolytic cleavage sites display backbone flexibility in both Bet v 1 isoforms and (ii) Bet v 1.0102 is consistently the more flexible protein in all cases. In addition, the hydrogen-deuterium exchange data show that backbone amides at or close to the early cleavage sites are more solvent exposed in Bet v 1.0102 compared to Bet v 1.0101, in line with proteolysis studies indicating that Bet v 1.0102 is more susceptible to proteolysis [12,13]. Our observations may thus explain the different proteolytic behavior of these isoforms and suggest that backbone flexibility could indeed be important in promoting proteolytic cleavage. The backbone accessibility and flexibility of an antigen’s polypeptide chain at the site of proteolytic attack are likely to promote optimal binding and proper interaction with proteases.

Correlations between antigen flexibility and proteolytic cleavage have been observed in previous studies [34]. Carmicle et al. showed that Hsp10 proteins have a strong preference for proteolytic cleavage in conformationally flexible loops [36]. The replacement of flexible loops by tight turns eliminated the protease sensitivity of these antigens and decreased their immunogenicity, suggesting that the conformation of the polypeptide around the cleavage site must be easily adjustable in order to bind to the protease [37]. Along the same lines, Wijesinha-Bettoni et al. proposed that the limited conformational flexibility of nonspecific lipid transfer proteins (LTPs) is responsible for this antigen’s high resistance to proteolysis [38]. For food allergens like LTPs, resistance to gastroduodenal digestion is a key component of their allergic potential. Using molecular dynamics simulations and NMR hydrogen-deuterium exchange experiments, it was shown that this protein remains tightly folded and compact with a rigid backbone, even at low pH values. Notably, an increased level of structural flexibility was found for peach LTP, in accordance with the slightly elevated susceptibility to proteolysis of this specific antigen. The authors concluded that a certain degree of flexibility is required for the interacting of LTPs with proteases, presumably also involving side chains of interacting residues [38]. Moreover, molecular dynamics simulations indicate that lipid binding to wheat LTP enhances the side chain mobility at various sites and increases the susceptibility of the polypeptide backbone to proteolytic cleavage [39]. Likewise, for the highly stable peanut 2S albumins Ara h 2 and Ara h 6, proteolytic cleavage sites are limited to polypeptide segments that are conformationally highly flexible, as suggested by MD simulations [40].

To sum up, we have shown that highly homologous isoforms of the major birch pollen allergen can have strikingly different features when it comes to conformational flexibilities. Since a certain level of flexibility is required for the proteolytic cleavage of buried peptide bonds, the differences between two Bet v 1 isoforms that we observe may well represent a molecular fingerprint of the proteolytic processing characteristics of these proteins. For a comprehensive description of the interplay between the conformational flexibility and proteolytic processing of Bet v 1 isoforms, future NMR measurements and MD simulations under conditions that mimic the physiologically different stages of the endolysosome will be essential.

## 4. Materials and Methods

### 4.1. NMR Sample Preparation

Protein expression pET28b vectors encoding Bet v 1.0101 (UniProt P15494) and Bet v 1.0102 (UniProt P43177) were kindly provided by Fatima Ferreira (University of Salzburg, Salzburg, Austria). Uniformly ^15^N-labeled Bet v 1 proteins were isolated and purified from *Escherichia coli* strain BL21(DE3) Star cultures grown in minimal M9 media enriched with ^15^NH_4_Cl at 310 K (for Bet v 1.0101) and 289 K (for Bet v 1.0102). The lower expression temperature for isoform Bet v 1.0102 was chosen to optimize yields, while avoiding the formation of inclusion bodies [41]. Purification was performed as described for Bet v 1.0101 [26] and Bet v 1.0102 [42]. Briefly, both procedures contain a hydrophobic interaction chromatography step using 3 × 5 mL HiTrap™ Phenyl FF columns (GE Healthcare Life Sciences, Uppsala, Sweden), followed by a final size-exclusion chromatography using a 16/60 Superdex75 prep grade column (GE Healthcare Life Sciences, Uppsala, Sweden) with 5 mM sodium phosphate at pH 8.0 as the running buffer. Protein concentrations were determined using a Nano-Photometer Pearl (Implen, Munich, Germany) with an extinction coefficient, ε_0_, of 1,0430 M^−1^ cm^−1^.

### 4.2. NMR Experiments and Data Analysis

^15^N relaxation dispersion experiments were recorded at 298 K on a Varian Inova 800 MHz spectrometer equipped with a room-temperature probe, using the Carr-Purcell-Meiboom-Gill (CPMG) pulse sequences described previously [43,44]. All data were processed using NMRPipe [45]. The experiments were performed using samples containing 0.4 mM Bet v 1 protein in 5 mM sodium phosphate pH 8.0 buffer and 8% D_2_O. Spectra were collected as series of data sets with CPMG field strengths, ν_CPMG_ = 1/(2·*T*_CPMG_), where *T*_CPMG_ is the time between two successive 180° pulses in the CPMG pulse train with a length of *T*_relax_ = 30 ms, between 33 and 933 Hz (with repeat experiments at 67 and 600 Hz), with a relaxation delay set to 30 ms. Spectra were recorded as 1366 × 60 complex points with a maximum acquisition time of 50 ms in the ^15^N dimension. The *t*_1_ (*t*_2_) domain data were apodized using shifted sine bell functions in both dimensions and zero-filled to 512 (2048) data points. Partial peak volumes were obtained by adding the intensities in 5 × 5 grids centered on the peak maximum, and converted to effective relaxation rates via *R*_2,eff_ = −1/*T*_relax_·ln(*I*/*I*_0_), where *I* is the partial peak volume at a given CPMG field strength and *I*_0_ is the partial peak volume in a reference experiment recorded without *T*_relax_. The relaxation dispersion data were analyzed by globally fitting the Carver-Richards equation [46] to the experimental data using in-house-written software.

Backbone amide hydrogen-deuterium exchange experiments were performed at 283 K on a Varian DirectDrive2 500 MHz spectrometer equipped with a room-temperature probe. To initiate H/D hydrogen-deuterium exchange, ^15^N labeled Bet v 1 in 5 mM sodium phosphate pH 8.0 buffer (0% D_2_O) was added to an equal volume of 5 mM sodium phosphate pH 8.0 buffer (100% D_2_O), to a final concentration of 0.4 mM Bet v 1. Immediately after sample preparation, a series of two-dimensional SOFAST spectra were recorded as previously described [21], using 1026 × 80 complex points, each lasting 8.5 min. The *t*_1_ (*t*_2_) domain data were apodized using shifted sine bell functions, zero-filled to 256 (4096) data points, and partial peak volumes were obtained by adding the intensities in 5 × 5 grids. The hydrogen-deuterium exchange data were analyzed by individually fitting exponential decays to the experimental data for each residue.

### 4.3. Molecular Dynamics Simulations

Molecular dynamics simulations of Bet v 1 isoforms were performed using the AMBER14 simulation package [47]. For Bet v 1.0101, the crystal structure with the Protein Data Bank (PDB) accession code 4A88 [18] was used as the starting point, while the starting structure for Bet v 1.0102 was modeled based on 4A88 by the introduction of seven point mutations, rotamer exploration of mutated residues, and subsequent local energy minimization. Structures were protonated using the protonate3D tool [48] as implemented in MOE (Molecular Operating Environment, Chemical Computing Group, version 2014.0901, Montreal, Canada) [49]. Systems were soaked into a truncated octahedral solvent box of TIP3P water molecules [50] with a minimum wall distance of 12 Å. Protein atoms were described using the Amber force field ff99SB-ILDN [51] with hydrogen bond lengths constrained by applying the SHAKE algorithm [52]. After applying an elaborated equilibration protocol including multiple energy minimization, and heating and cooling steps [53], systems were sampled in the NpT ensemble for 1 µs using the graphics processing unit (GPU) implementation of pmemd [54] and a time step of 2.0 fs. Two independent simulations were performed for each system at a 300 and 360 K simulation temperature, maintained via a Langevin thermostat [55]. The resulting trajectories were stored as 50,000 equal-spaced snapshots and analyzed using cpptraj from Ambertools [56], along with in-house scripts. After ensuring the structural and energetic stability, we calculated the ensemble-averaged radii of gyration for Cα atoms, total intramolecular hydrogen bonds (using default cut-offs), and secondary structure contents for all Bet v 1 isoforms. To characterize the dynamic properties, we extracted residue-wise B-factors for Cα atoms after performing a global alignment to the starting structure [57].

## Figures and Tables

**Table 1:** Molecular dynamics simulations of nanosecond-microsecond flexibility in Bet v 1 isoforms. Comparison of structural and dynamical descriptors extracted from the conformational ensembles in 300 and 360 K MD simulations.

	Bet v 1.0101	Bet v 1.0102	Bet v 1.0101	Bet v 1.0102
*T* (K)	300.0	300.0	360.0	360.0
*SASA* (Å^2^) ^a^	8210	8338	8376	8607
*nHB* ^b^	82.7	78.5	76.5	75.4
Avg. *B* (Å^2^) ^c^	20.8	34.6	48.3	92.5

^a^ *SASA*, solvent accessible surface area; ^b^ *nHB*, average number of hydrogen bonds; ^c^ avg *B*, average *B*-factor.
